# Multisensory Home-Monitoring in Individuals With Stable Chronic Obstructive Pulmonary Disease and Asthma: Usability Study of the CAir-Desk

**DOI:** 10.2196/31448

**Published:** 2022-02-16

**Authors:** Dario Kohlbrenner, Christian F Clarenbach, Adam Ivankay, Lukas Zimmerli, Christoph S Gross, Manuel Kuhn, Thomas Brunschwiler

**Affiliations:** 1 Department of Pulmonology University Hospital Zurich Zurich Switzerland; 2 Faculty of Medicine University of Zurich Zurich Switzerland; 3 IBM Research Europe - Zurich Zurich Switzerland; 4 Department of Management, Technology, and Economics ETH Zurich Zurich Switzerland

**Keywords:** home monitoring, digital health, respiratory disease, usability, feasibility, adherence, disease management, chronic disease, patient monitoring

## Abstract

**Background:**

Research integrating multisensory home-monitoring in respiratory disease is scarce. Therefore, we created a novel multisensory home-monitoring device tailored for long-term respiratory disease management (named the CAir-Desk). We hypothesize that recent technological accomplishments can be integrated into a multisensory participant-driven platform. We also believe that this platform could improve chronic disease management and be accessible to large groups at an acceptable cost.

**Objective:**

This study aimed to report on user adherence and acceptance as well as system functionality of the CAir-Desk in a sample of participants with stable chronic obstructive pulmonary disease (COPD) or asthma.

**Methods:**

We conducted an observational usability study. Participants took part in 4 weeks of home-monitoring with the CAir-Desk. The CAir-Desk recorded data from all participants on symptom burden, physical activity, spirometry, and environmental air quality; data on sputum production, and nocturnal cough were only recorded for participants who experienced symptoms. After the study period, participants reported on their perceptions of the usability of the monitoring device through a purpose-designed questionnaire. We used descriptive statistics and visualizations to display results.

**Results:**

Ten participants, 5 with COPD and 5 with asthma took part in this study. They completed symptom burden questionnaires on a median of 96% (25th percentile 14%, 75th percentile 96%), spirometry recordings on 55% (20%, 94%), wrist-worn physical activity recordings on 100% (97%, 100%), arm-worn physical activity recordings on 45% (13%, 63%), nocturnal cough recordings on 34% (9%, 54%), sputum recordings on 5% (3%, 12%), and environmental air quality recordings on 100% (99%, 100%) of the study days. The participants indicated that the measurements consumed a median of 13 (10, 15) min daily, and that they preferred the wrist-worn physical activity monitor to the arm-worn physical activity monitor.

**Conclusions:**

The CAir-Desk showed favorable technical performance and was well-accepted by our sample of participants with stable COPD and asthma. The obtained insights were used in a redesign of the CAir-Desk, which is currently applied in a randomized controlled trial including an interventional program.

## Introduction

Respiratory disease has a huge impact on global health. Taken together, both acute and chronic respiratory disease rank second among all-cause mortality [1]. At the same time, chronic respiratory disorders carry substantial disease burden. Chronic obstructive pulmonary disease (COPD), a major chronic respiratory condition, poses a substantial risk of disability (the ninth most frequent cause of disability worldwide) [1]. Global networks of experts engage in improving diagnostic approaches and researching the most effective treatment modalities for chronic respiratory disease [2,3]. Current evidence indicates multimodal diagnostic and treatment approaches to be the method of choice.

Technological advances substantially impact health care. Home-monitoring and self-reporting allow more frequent measurements of individuals’ symptoms and behaviors, supported by seamless data transfer, storage, and analysis in the cloud, enabling on-demand overviews of subjects’ health states and trajectories. However, cutting-edge technology seems scarcely implemented into clinical practice and clinical research. The most widely used approach is “tele-healthcare,” in which part of the management is carried out through telephone calls [4-7]. This is an interesting option in mobility-compromised populations or for minor health issues. However, this approach is still staff-intense and provides a unidimensional focus on solely patient-reported outcomes (PROMs). Furthermore, appointments, and accordingly the time points when the PROMs are measured, are most commonly dictated by the schedule of the health care professionals and not by the time point at which participants experience symptoms, attacks, barriers, or insecurities. We hypothesize that recent technological accomplishments can be integrated into a multisensory participant-driven platform, which involves both PROMs and disease markers incorporating multiple measures from a variety of sensors. We also believe that this platform could improve chronic disease management and be accessible to large groups at an acceptable cost.

Research integrating multisensory home-monitoring in respiratory disease is scarce [8]. The available investigations were most commonly unidimensional, short-term, and small-scale. To overcome these shortcomings, we developed a novel multisensory home-monitoring device tailored for long-term respiratory disease management (named the “CAir-Desk”), which is optimized for user experience, health workflow, and outcomes. In this study, we report on system functionality of the novel CAir-Desk and feasibility, as well as user adherence and acceptance in a sample of participants with stable COPD or asthma.

## Methods

### Study Design

**Figure 1:**
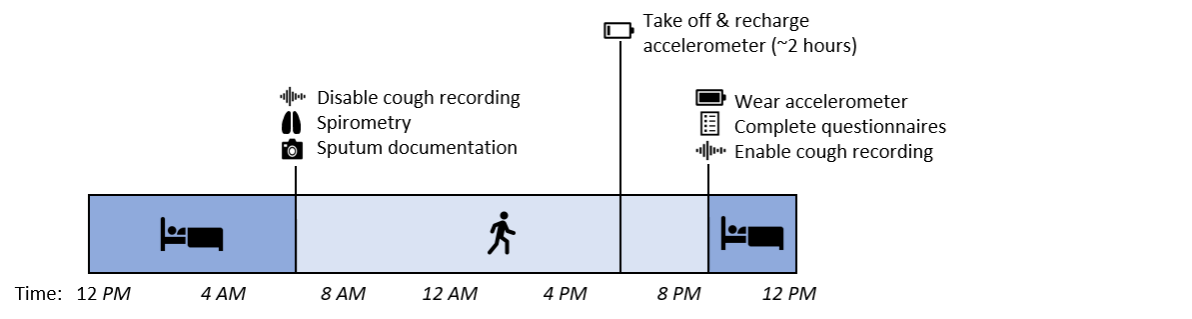
Daily measurement schedule during the study period.

We conducted an observational usability study. Participants conducted 4 weeks of disease home-monitoring with the CAir-Desk, without interventions or modifications to their established treatment regimen. The participants were instructed to place the CAir-Desk in their bedroom and take daily measurements in accordance with a schedule (Figure 1). After the study period, participants reported their perceptions on the usability of the CAir-Desk with a purpose-designed questionnaire (Multimedia Appendix 1). This study did not fall within the scope of the Human Research Act (HRA) [9] and did not require authorization from the ethics committee. The Ethics Committee of the Canton of Zurich confirmed this in the BASEC Request (2018-00180).

### Study Participants

We used convenience sampling for this study. Ten participants with stable COPD or asthma, attending outpatient secondary care at the Department of Pulmonology, University Hospital Zurich, Switzerland, consented to take part in the study.

### The CAir-Desk

**Figure 2:**
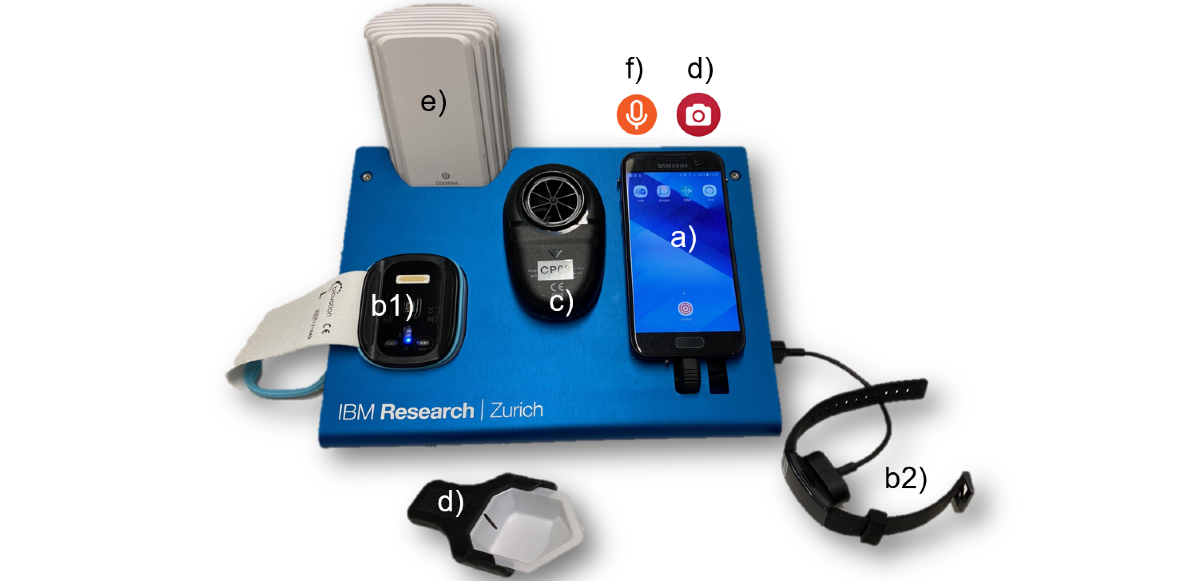
The CAir-Desk setup for the usability study as a collection of sensors. Items indicated here are as follows: (a) smartphone, (b1) arm-worn accelerometer, (b2) wrist-worn accelerometer, (c) spirometer, (d) sputum collector and smartphone camera, (e) environmental air quality monitor, and (f) nocturnal cough monitor.

The CAir-Desk (Figure 2), is a novel, custom-built disease home-monitoring system [10]. It combines multiple sensors in a compact format with a single power plug for device charging. All components of the CAir-Desk are conformité européenne (CE)-certified.

#### Smartphone

The smartphone (Galaxy A320, 2017, Samsung Group)—object *a* in Figure 2—contained purpose-designed apps for user interaction and data visualization. All sensors are accessible via the smartphone. Further, all sensor data are transferred to the cloud storage through the smartphone by the Global System for Mobile Communications (GSM) network.

#### Physical Activity

Physical activity was measured using multisensory triaxial accelerometry (Charge 3, Fitbit Inc, and Everion, Biovotion). We decided to include 2 physical activity monitors for this usability study to conclude on the participants’ preference. The Charge 3 is a wrist-worn device (item *b2* in Figure 2) with a small display that provides real-time information on step count. The Everion does not include a display and is worn on the upper arm, (item *b1* in Figure 2). It only provides information when synchronized with the CAir-Desk.

#### Symptom Burden

Symptom burden in the participants diagnosed with COPD was assessed using the COPD Assessment Test [11]. For the participants diagnosed with asthma, a purpose-designed questionnaire was used (Multimedia Appendix 2). The questionnaires were sent out through the patient-provider communication channel app (docdok.health). The smartphone displayed push notifications when the daily questionnaire was due, and participants answered the questions directly in the app.

#### Nocturnal Cough

Nocturnal cough recordings were collected using the smartphone microphone (item *f* in Figure 2). The participants had to manually enable recording every night before going to bed and turn it off in the mornings. A purpose-designed algorithm isolated and extracted the cough count from the background noise. The CAir-Desk app displayed cough count in running monitoring sessions in near real-time (10-second delay) [12]. This was a voluntary measurement; we encouraged only participants with a self-reported cough to use the sensor.

#### Spirometry

Daily spirometry recordings were obtained with a portable spirometry device (Air Next Spirometer, NuvoAir)—item *c* in Figure 2—which connected with the smartphone via Bluetooth. The values obtained were forced expiratory volume in 1 second (FEV_1_) and forced vital capacity (FVC). All participants were trained to produce reproducible maneuvers that comply with published guidelines [13].

#### Sputum Monitoring

This was a voluntary measurement; we encouraged only those participants with self-reported sputum production to submit a photograph of a sample to the system. This was accomplished using the built-in smartphone camera and purpose-designed sputum collectors (item *d* in Figure 2).

#### Environmental Air Quality

The CAir-Desk continuously recorded environmental air quality (Foobot, Airboxlab)—item *e* in Figure 2. The values obtained were temperature, humidity, particulate matters, and volatile organic compounds. No interaction with the CAir-Desk was needed from the participants to enable data-recording.

#### Cloud and Backend Setup

Data transfer to and data storage on the cloud were performed with encryption, as required by data protection regulations for sensitive personal information.

Further details about technical, cloud, and backend solutions can be found elsewhere [10].

#### Device Usage and Adherence Thresholds

To identify days of adherence, we defined individual thresholds for each sensor in accordance with the study protocol. An overview of these thresholds for each sensor, together with details on recording modalities, is provided in Table 1.

**Table 1:** Overview of the sensor and adherence thresholds.

Sensor		Relevant modalities	Adherence day threshold
**Mandatory^a^**			
	Physical activity, wrist-worn	Step count	Steps ≥ 100
	Physical activity, arm-worn	Step count	Recording ≥ 1 hour
	Symptom burden	CAT^b^ [11] or Asthma questionnaire	Completed questionnaire
	Spirometry	Forced expiratory volume in 1 second, forced vital capacity	≥3 valid exhalations
	Environmental air quality	Volatile organic compounds, temperature	Recording ≥ 1 hour
**Voluntary^c^**			
	Nocturnal cough	Audio (.wav) file count	Recording ≥ 1 audio file
	Sputum	Photo count	Recording ≥ 1 photo

^a^Measurements were requested from all participants.

^b^CAT: chronic obstructive pulmonary disease assessment test.

^c^Measurements were requested from participants experiencing specific symptoms.

### Statistical Analysis

This small-scale usability study did not allow assumptions on normally distributed data, which we confirmed visually using quantile-quantile plots. The day of instruction and the day of return of the CAir-Desk were both excluded from the analysis, only considering full study days. In case of technical issues, for example, when a sensor was not recording for the whole or part of the study period, these data were excluded from adherence-reporting.

We extracted and prepared the data for statistical analysis with Python 3.7.10 (The Python Software Foundation, 2021). We analyzed the data statistically with R 4.0.3 (R Core Team, 2021, R Foundation for Statistical Computing).

## Results

All results are presented using descriptive statistics in the format median (25th percentile, 75th percentile) unless otherwise stated.

Ten participants, 5 with COPD (61 [59, 62] years; median FEV_1_ predicted=69% [60%, 78%]) and 5 with asthma (55 [55, 55] years; median FEV_1_ pred=62% [62%, 69%]) were included in this study (detailed characteristics in Table 2) and used the CAir-Desk for 27.5 (27, 28) days, excluding the on- and off-boarding days. All participants completed the predetermined study period and did not experience any adverse events.

**Table 2:** Study participant characteristics stratified in accordance with their diagnosis.

Characteristics	Participants with chronic obstructive pulmonary disease (n=5)	Participants with asthma (n=5)
Age (years), median (25th percentile, 75th percentile)	61 (59, 62)	55 (55, 55)
Sex (female/male), n (%)	3/2 (60/40)	1/4 (25/75)
Forced expiratory volume in 1 second (% predicted), median (25th percentile, 75th percentile)	69 (60, 78)	62 (62, 69)
Smoking status (yes/no), n (%)	1/4 (25/75)	0/5 (0/100)

### Participant Adherence and Technical Considerations

#### Questionnaires

**Figure 3:**
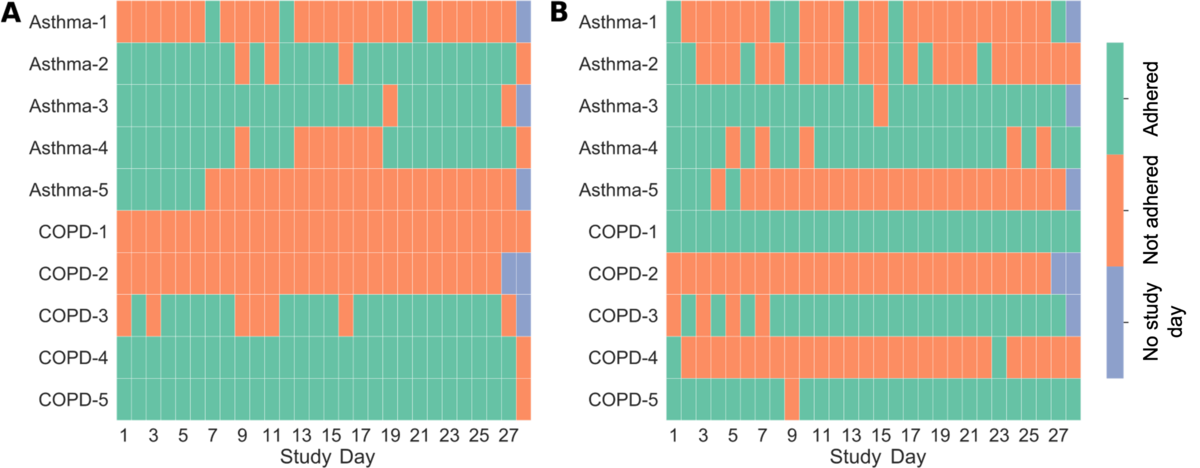
Individual daily adherence for symptom burden questionnaires (A) and spirometry (B). All participants are shown (ie, including those with nonadherence due to technical difficulties). COPD: chronic obstructive pulmonary disease.

**Figure 4:**
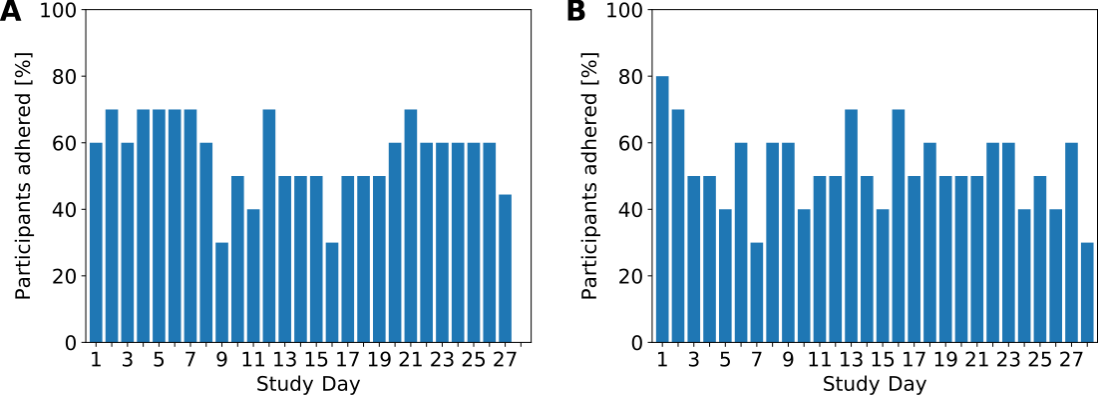
Daily adherence (ratio of participants who adhered to the total number of study participants) for symptom burden questionnaires (A) and spirometry (B). All participants are represented (ie, including those with nonadherence due to technical difficulties).

In total, participants completed 96% (14%, 96%) of the daily questionnaires. Participants with COPD completed 96% (0%, 96%) and those with asthma completed 96% (22%, 96%) of the daily questionnaires. Owing to a technical issue, one participant with COPD did not receive the questionnaires and was excluded from the analysis of adherence data. Figure 3A provides individual adherence rates throughout the study period; Figure 3A indicates participants who could not adhere owing to technical difficulties. Except for 2 days, adherence rates with respect to the study days remained constantly high throughout the study period (Figure 4A).

#### Spirometry

Participants completed valid spirometry recordings (ie, 3 or more attempts) on 55% (20%, 94%) of the study days. Participants with COPD completed valid spirometry recordings on 96% (48%, 96%) and participants with asthma completed recordings on 29% (22%, 82%) of the study days. For 2 participants with COPD, the data upload partially failed during the study period. These participants were excluded from the analysis of adherence data. Figure 3B shows individual adherence rates throughout the study period; Figure 3B includes participants who could not adhere owing to technical difficulties. There was variability in daily adherence rates throughout the study period with more consistency toward the end of the study (Figure 4B).

#### Physical Activity

Participants wore the wrist-worn accelerometer on 100% (97%, 100%) of the study days. Participants with COPD wore the wrist-worn accelerometer on 100% (100%, 100%) and those with asthma wore it on 100% (96%, 100%) of the study days.

Participants wore the arm-worn accelerometer on 45% (13%, 63%) of the study days. Participants with COPD wore the arm-worn accelerometer on 61% (30%, 80%) and those with asthma wore it on 30% (14%, 63%) of the study days. For 2 participants with COPD, the arm-worn accelerometer broke during daily use and could not be used further; these participants were excluded from the analysis of adherence data.

#### Nocturnal Cough Recording

Participants enabled nocturnal cough recording on 34% (9%, 54%) of the study nights. Participants with COPD enabled nocturnal cough–recording on 46% (23%, 59%) and those with asthma enabled it on 22% (11%, 50%) of the study nights. For 2 participants with COPD, the data upload did not proceed as expected throughout part of the study period; these participants were excluded from the analysis of adherence data.

#### Sputum Monitoring

Participants took photographs of their sputum on 5% (3%, 12%) of the study days. Participants with COPD took photographs of their sputum on 4% (2%, 16%) and those with asthma on 7% (4%, 11%) of the study days. For 2 participants with COPD, the data upload did not proceed as expected throughout part of the study period; these participants were excluded from the analysis of adherence data.

#### Environmental Air Quality Monitoring

Participants measured environmental air quality on 100% (99%, 100%) of the study days. Participants with COPD measured environmental air quality on 100% (100%, 100%) and those with asthma on 100% (96%, 100%) of the study days. For 2 participants with COPD, the data upload did not proceed as expected during part of the study period; these participants were excluded from the analysis of adherence data. One participant with asthma turned off the data transfer connection for the sensor by accident for a short time, which resulted in 15 days of nonrecording. We retained this participant in the analysis.

**Figure 5:**
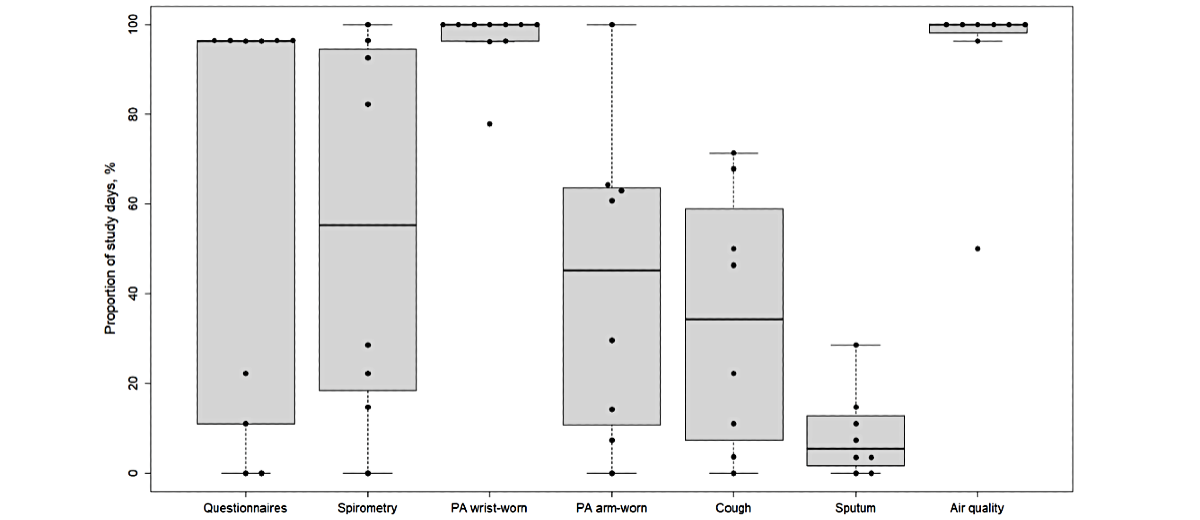
Adherence stratified for sensors. Data are the percentages of study days. PA, physical activity.

Adherence data on the questionnaires, spirometry recordings, physical activity, nocturnal cough recording, sputum monitoring, and environmental monitoring are presented on individual and summary levels in Table 3 and are visualized in Figure 5.

**Table 3:** Sensor adherence data on a participant^a^ and summary^b^ level.

Participant number	Diagnosis	Mandatory^c^						Voluntary^d^	
		Questionnaire	Spirometry	Physical activity, wrist-worn	Physical activity, arm-worn	Air quality	Nocturnal cough		Sputum
1	Asthma	11	22	78	63	96	11		7
2	Asthma	96	29	100	14	50	68		0
3	Asthma	96	93	100	30	100	4		11
4	Asthma	96	82	100	64	100	50		4
5	Asthma	22	15	96	7	100	22		15
**Summary of participants with asthma**	N/A^e^	96 (22, 96)	29 (22, 82)	100 (96, 100)	30 (14, 63)	100 (96, 100)	22 (11, 50)		7 (4, 11)
6	COPD^f^	0	100	100	61	100	46		4
7	COPD	N/A	0	96	100	100	0		0
8	COPD	96	N/A	100	N/A	N/A	N/A		N/A
9	COPD	96	N/A	100	N/A	N/A	N/A		N/A
10	COPD	96	96	100	0	100	71		29
**Summary of participants with COPD**	N/A	96 (0, 96)	96 (48, 98)	100 (100, 100)	61 (30, 80)	100 (100, 100)	46 (23, 59)		4 (2, 16)
**Summary total**	N/A	96 (14, 96)	55 (20, 94)	100 (97, 100)	45 (13, 63)	100 (99, 100)	34 (9, 54)		5 (3, 12)

^a^Individual data are percentage values of the total number of study days.

^b^Summary data are median (25th percentile, 75th percentile) percentage values of the total number of study days.

^c^Measurements were obtained from all participants.

^d^Measurements were only requested from participants who experienced specific symptoms.

^e^N/A: not applicable.

^f^COPD: Chronic obstructive pulmonary disease.

### Participant Satisfaction

Regarding the CAir-Desk, in general, 6 (60%) participants indicated that they would be willing to use the device further as is, and 1 (10%) participant indicated that he would not. Furthermore, 2 (20%) participants indicated a neutral response, and 1 participant (10%) did not answer the question. Overall, participants reported to have taken a median of 13 (10, 15) minutes per day manipulating the device, which 7 (70%) participants considered an acceptable amount of time, 2 (20%) participants considered too much time, and 1 (10%) participant indicated a neutral response.

#### Questionnaires

Concerning further use, 4 (40%) participants indicated that they would be willing to continue daily reporting, and 4 (40%) participants indicated that they would not. Two (20%) participants indicated a neutral response.

Regarding user-friendliness, 4 (40%) participants considered the questionnaires user-friendly, and 6 (60%) did not.

#### Spirometry

Regarding further use, 4 (40%) participants indicated that they would be willing to continue daily recordings, and 3 (30%) participants indicated that they would not. Two (20%) participants indicated a neutral response, and 1 (10%) participant did not use the spirometry device.

Regarding user-friendliness, 7 (70%) participants considered the spirometry device user-friendly, and 3 (30%) did not.

#### Physical Activity

Regarding further use of the wrist-worn accelerometer, 9 (90%) participants indicated that they would be willing to continue wearing the device, and 1 (10%) participant indicated that he would not.

Regarding user-friendliness, 9 (90%) participants considered the wrist-worn accelerometer user-friendly and 1 (10%) did not.

Regarding further use of the arm-worn accelerometer, 5 (50%) participants indicated that they would be willing to continue wearing the device, and 4 (40%) indicated that they would not. Furthermore, 1 (10%) participant indicated a neutral response.

Regarding user-friendliness, 5 (50%) participants considered the arm-worn accelerometer user-friendly and 5 (50%) did not.

#### Nocturnal Cough Recording

Regarding further use, 4 (40%) participants indicated that they would be willing to continue recordings, and 4 (40%) indicated that they would not. Furthermore, 2 (20%) participants indicated a neutral response.

Regarding user-friendliness, 4 (40%) participants considered the nocturnal cough recording user-friendly and 6 (60%) did not.

#### Sputum Monitoring

Regarding further use, 3 (30%) participants indicated that they would be willing to continue taking photographs, and 2 (20%) participants indicated that they would not. Furthermore, 2 (20%) participants indicated a neutral response, and 2 (20%) did not use this function owing to no sputum production. One (10%) participant did not answer this question.

Regarding user-friendliness, 3 (30%) participants considered sputum monitoring user-friendly and 7 (70%) did not.

#### Environmental Air Quality Monitoring

Regarding further use, 8 (80%) participants indicated that they would be willing to continue air quality monitoring, and 2 (20%) indicated a neutral response.

Regarding user-friendliness, 5 (50%) participants considered environmental air quality monitoring user-friendly and 5 (50%) did not.

**Figure 6:**
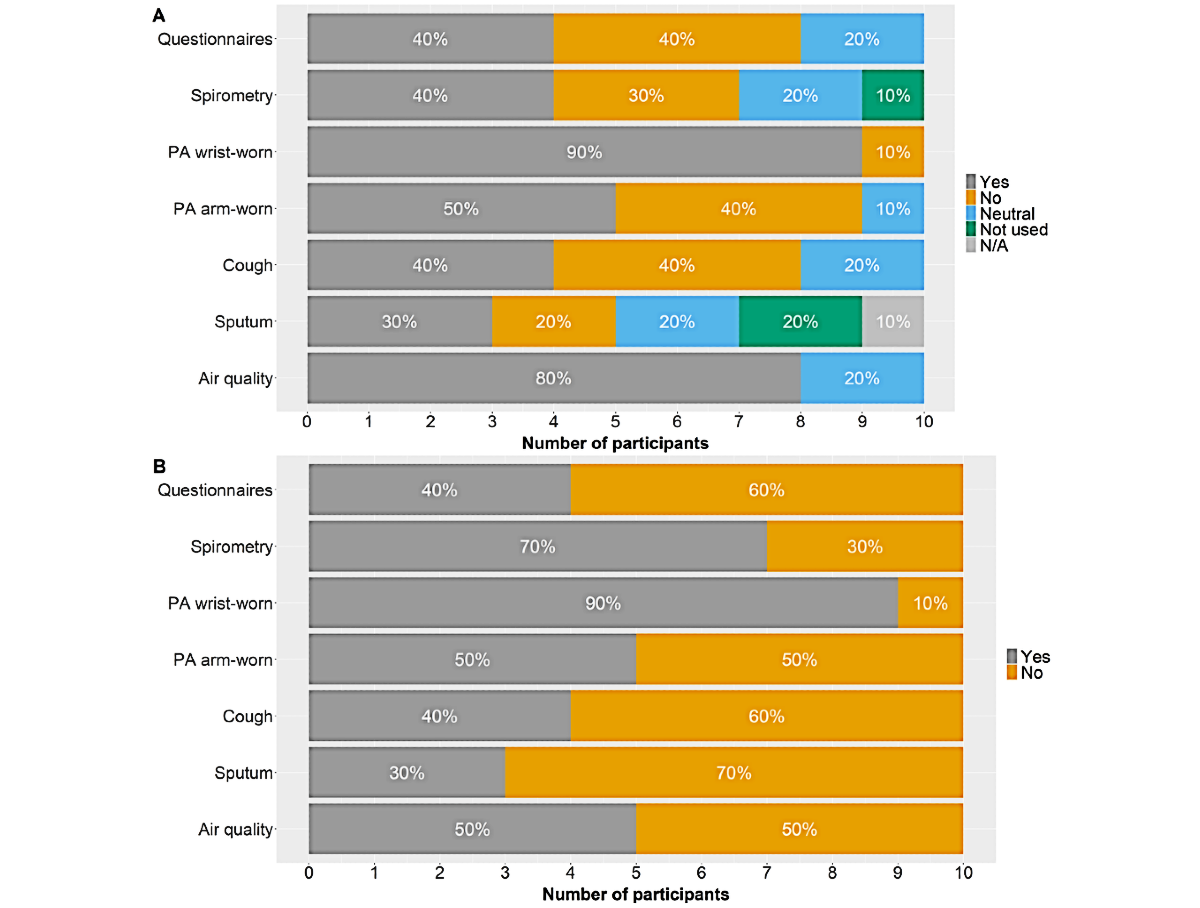
Participant satisfaction data stratified for sensors. (A) “Would you be willing to use [insert sensor] further?”. (B) “Do you consider [insert sensor] as user-friendly?” Data are n (on the x-axis) and % (on the bars). PA: physical activity.

**Figure 7:**
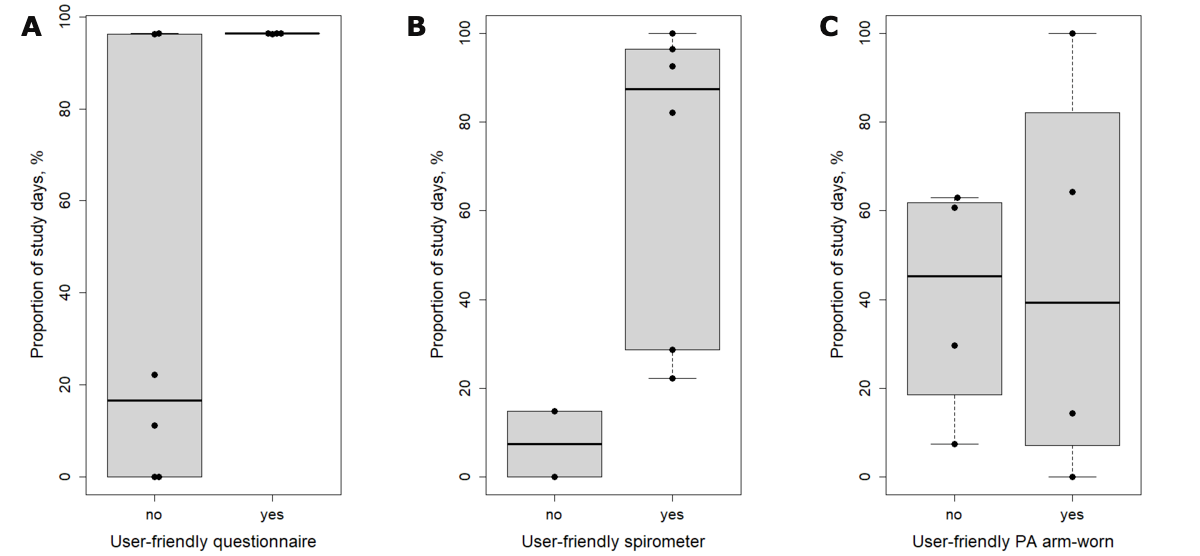
Adherence stratified according to the user-friendliness of sensors. Questionnaires (A), spirometry (B), arm-worn accelerometer (C). Data are the percentages of study days. PA: physical activity.

Participant satisfaction data on the questionnaires, spirometry, physical activity, nocturnal cough recording, sputum monitoring, and environmental monitoring are displayed in Figure 6. Adherence to questionnaires, spirometry, and physical activity monitoring stratified in accordance with the participants’ rating on user-friendliness (Figure 7).

## Discussion

### Principal Findings

We report on the first clinical application of the CAir-Desk. In this usability study, the CAir-Desk performed well technically and was well-accepted by our sample of participants with stable COPD and those with asthma.

### General Considerations

In general, participants indicated that they would be willing to use the CAir-Desk further. Since this was a purely observational study, we conclude that participants, although in stable phases of COPD or asthma, are interested in disease monitoring. The time consumption that the participants reported was lower than expected and, for the vast majority (80%), did not exceed their personal tolerance. We consider this a positive finding, since we performed the usability study in preparation for a subsequent randomized controlled trial (RCT) on the effectiveness of delivering “Living well with COPD” by a chatbot through the CAir-Desk, in combination with the home-monitoring [10,14]. When receiving an intervention through the CAir-Desk, time spent manipulating the device will certainly increase. However, we expect the amount of time to still be acceptable given our present findings.

General adherence to the intense measurement schedule was considerably high. Inconsistency in adherence rates between individuals was observed in spirometry and nocturnal cough measurements, which was due to the voluntary nature of these measurements.

Considering that this was the first clinical application of the CAir-Desk, technical performance and robustness was satisfactory. We faced problems when transferring data to the cloud with 2 participants, which were resolved remotely (however, leading to incomplete adherence data). Regarding hardware robustness, participants reported 2 broken arm-worn accelerometers during the study time. These had to be replaced and reconnected to the CAir-Desk by study staff, which led to an unplanned additional study visit and incomplete adherence data.

In response to the in-field testing, the CAir-Desk was updated for the subsequent RCT by including a script, which opens the relevant sensor apps daily, to guarantee data synchronization even when apps are closed unintentionally. Furthermore, we decided to use the wrist-worn accelerometer in any further clinical application of the CAir-Desk.

### Detailed Considerations

Regarding the daily questionnaires that the participants were asked to answer, we found high adherence, although the willingness for further use and user-friendliness did not receive consistently high ratings. One participant was not able to answer the questionnaires because the font was too small for him to read. For this usability study, we recruited individuals with stable COPD or asthma. Therefore, we hypothesize that daily administration of questionnaires might be too frequent since only minor daily variation in symptoms and general health status are expected. However, we would still recommend daily questionnaires in samples that are not in a stable disease phase, receive a new intervention, or are newly diagnosed. We recommend using a short questionnaire, keeping time consumption low and adherence high. Finally, large fonts or zoom functionality on the questionnaires is crucial to allow completion for individuals with compromised vision. Since this is a feasibility study investigating a very small sample, some caution on the interpretation is needed. This applies especially to the questionnaires in the case of the participants with COPD, since conclusive data are available from only three participants.

Regarding spirometry, we observed high interindividual variability. While one participant with COPD never took a test, another participant adhered strictly to the schedule. Overall, the adherence with respect to study days was almost constant across the study period (Figure 4B). Unfortunately, upload problems in 2 participants prevented us from drawing stronger conclusions on adherence patterns. However, we consider acceptance to daily spirometry tests as high, with only 2 participants reporting that they would not be willing to perform further tests. Similar to the questionnaires, we hypothesize that individuals with unstable disease or recent diagnosis would be more interested in daily measurements.

Regarding physical activity monitoring, we integrated two different devices for this usability study. We were interested if the participants preferred the arm-worn device or the wrist-worn device. The main differences between the devices are visibility to others, live access to measurements through a built-in display, and battery life. Participants clearly preferred the wrist-worn device (with built-in display and 5-day battery life instead of 1 day for the arm-worn device), which was also reflected in adherence rates. Only one participant reported the wrist-worn accelerometer not to be user-friendly, because the display turned on during sudden movements at night and caused awakenings. From a technical point of view, both devices can record heart rate. However, validity is low and may not yet serve medical purposes [15].

Regarding nocturnal cough recording, adherence was highly variable because only participants experiencing cough were encouraged to take measurements. User-friendliness was considered low. Participants had to turn on the cough counter manually every time they went to bed and turn it off when they got up. The majority of the participants considered this not user-friendly and we therefore updated the application following the study and added the feature for automated recording during individually tailorable time periods.

Regarding sputum monitoring, only the few participants who were experiencing sputum production were encouraged to take measurements. Again, we consider the option to submit a photograph of sputum samples as valuable in individuals with unstable disease. However, the CAir-Desk is nonportable; therefore, information on sputum production when individuals are not at home is missed. This has to be considered when populations are younger, and more active (eg, participants with cystic fibrosis).

Regarding environmental air quality monitoring, adherence was high, and participants indicated interest in further use. The device recorded automatically, and we only faced some days of nonrecording for 1 participant who accidentally turned off the Wi-Fi hotspot connection for several days. Unfortunately, the app displaying the results was only available in English. This fact led our participants, all German speakers, to low user-friendliness ratings.

Our findings suggest that missing data in similar studies to ours may be categorized into four categories: (1) missing owing to nonadherence (eg, did not like to use the sensor), (2) missing owing to technical issue (eg, failing upload or broken device), (3) missing owing to other issue (eg, allergy to worn device or impaired vision), (4) missing owing to absence of symptoms. Furthermore, it may be assumed that the participants who did not rate a sensor as user-friendly show lower adherence to that sensor. Our data show that this is not necessarily the case (Figure 7). While there was a clear trend to lower adherence in daily spirometry measurements among participants who did not consider the sensor user-friendly, a mixed response was observed in daily questionnaires, and an overall low adherence was observed in the arm-worn accelerometry measurements.

Considering the findings of this usability study, we modified the CAir-Desk, the corresponding sensors, apps, and the data upload. The modified version of the CAir-Desk is now used in an RCT delivering the multimodal intervention “Living well with COPD” through a chatbot [10].

### Conclusions

Our investigation indicates that multisensory disease monitoring using the CAir-Desk is feasible, and well accepted. Additionally, time consumption was surprisingly low and suggests that intervention delivery through the CAir-Desk can be performed with a reasonable time budget. Our results add knowledge on important points to consider when designing multisensory setups for individuals with chronic respiratory disease. First, sensors and apps should use automation as often as possible. Second, participants are interested in seeing their measurements and performance. Therefore, apps should grant easy interaction and provide an intuitive overview on the recorded information in the participant’s preferred language. Third, a rigid schedule with daily measurements is feasible and accepted by the participants. However, tailoring in accordance with the disease stage seems important to increase adherence in longer studies. Last, technical issues were rare and manageable.

We believe that the combination of home-based multisensory measurements and PROMs might be a game changer in chronic disease management. The frequent and accurate data obtained with the CAir-Desk might provide novel insights into early markers for a decline in health status or emergencies. We consider the combination of home-based measurements and information delivery combined with regular in-person care as the future of multimodal chronic disease management.

## Appendix

Multimedia Appendix 1 Usability questionnaire.

Multimedia Appendix 2 Asthma symptom burden questionnaire.

## Abbreviations

COPD: chronic obstructive pulmonary disease
FEV_1_: forced expiratory volume in 1 second
FVC: forced vital capacity
GSM: Global System for Mobile Communications
HRA: Human Research Act
PROM: patient-reported outcome
RCT: randomized controlled trial
