# Supplementary material for: Multisensory Home-Monitoring in Individuals With Stable Chronic Obstructive Pulmonary Disease and Asthma: Usability Study of the CAir-Desk
Source: JMIR Hum Factors. 2022 Feb 16;9(1):e31448. doi: 10.2196/31448 (PMC8892320; doi:10.2196/31448)
Supplement: Multimedia Appendix 1 [file humanfactors_v9i1e31448_app1.docx]

**Multimedia Appendix 1. Usability questionnaire.**

**Usability questionnaire at study termination**

Everion activity monitor:

- Synchronization of the Everion device worked properly.

❑ strongly agree

❑ agree

❑ neutral

❑ disagree

❑ strongly disagree

- Charging of the Everion device worked properly.

❑ strongly agree

❑ agree

❑ neutral

❑ disagree

❑ strongly disagree

- I got used to wearing the Everion device.

❑ strongly agree

❑ agree

❑ neutral

❑ disagree

❑ strongly disagree

- Would you be willing to use the Everion device further?

❑ strongly agree

yes

❑ agree

❑ neutral

❑ disagree

no

❑ strongly disagree

- It was interesting to monitor my physical activity with the Everion device.

❑ strongly agree

❑ agree

❑ neutral

❑ disagree

❑ strongly disagree

FitBit activity monitor:

- Synchronization of the Fitbit device worked properly.

❑ strongly agree

❑ agree

❑ neutral

❑ disagree

❑ strongly disagree

- Charging of the Fitbit device worked properly.

❑ strongly agree

❑ agree

❑ neutral

❑ disagree

❑ strongly disagree

- I got used to wearing the Fitbit device.

❑ strongly agree

❑ agree

❑ neutral

❑ disagree

❑ strongly disagree

- Would you be willing to use the Fitbit device further?

❑ strongly agree

yes

❑ agree

❑ neutral

❑ disagree

no

❑ strongly disagree

- It was interesting to monitor my physical activity with the Fitbit device.

❑ strongly agree

❑ agree

❑ neutral

❑ disagree

❑ strongly disagree

Spirometry:

- Measurements with the Nuvoair device worked properly.

❑ strongly agree

❑ agree

❑ neutral

❑ disagree

❑ strongly disagree

- I got used to the daily spirometry measurements.

❑ strongly agree

❑ agree

❑ neutral

❑ disagree

❑ strongly disagree

- Would you be willing to use the Nuvoair device further?

❑ strongly agree

yes

❑ agree

❑ neutral

❑ disagree

no

❑ strongly disagree

- It was interesting to monitor my lung function with the Nuvoair device.

❑ strongly agree

❑ agree

❑ neutral

❑ disagree

❑ strongly disagree

Environmental air-quality:

- Measurements with the Foobot device worked properly.

❑ strongly agree

❑ agree

❑ neutral

❑ disagree

❑ strongly disagree

- I did not mind having the Foobot in my bedroom.

❑ strongly agree

❑ agree

❑ neutral

❑ disagree

❑ strongly disagree

- Would you be willing to use the Foobot device further?

❑ strongly agree

yes

❑ agree

❑ neutral

❑ disagree

no

❑ strongly disagree

- It was interesting to monitor the air-quality with the Foobot device.

❑ strongly agree

❑ agree

❑ neutral

❑ disagree

❑ strongly disagree

Docdok health application:

- Measurements with the Questionnaire app worked properly.

❑ strongly agree

❑ agree

❑ neutral

❑ disagree

❑ strongly disagree

- Would you be willing to complete questionnaires on a daily basis further?

❑ strongly agree

yes

❑ agree

❑ neutral

❑ disagree

no

❑ strongly disagree

Nocturnal cough recording:

- Nocturnal cough recordings worked properly.

❑ strongly agree

❑ agree

❑ neutral

❑ disagree

❑ strongly disagree

- I got used to the nocturnal cough recordings.

❑ strongly agree

❑ agree

❑ neutral

❑ disagree

❑ strongly disagree

- Would you be willing to use the nocturnal cough recordings further?

❑ strongly agree

yes

❑ agree

❑ neutral

❑ disagree

no

❑ strongly disagree

- It was interesting to monitor my nocturnal cough.

❑ strongly agree

❑ agree

❑ neutral

❑ disagree

❑ strongly disagree

Sputum monitoring:

- Capturing photos of sputum samples worked properly.

❑ strongly agree

❑ agree

❑ neutral

❑ disagree

❑ strongly disagree

- I got used to take photos of my sputum samples.

❑ strongly agree

❑ agree

❑ neutral

❑ disagree

❑ strongly disagree

- Would you be willing to use the sputum photography further?

❑ strongly agree

yes

❑ agree

❑ neutral

❑ disagree

no

❑ strongly disagree

- It was interesting to monitor my sputum.

❑ strongly agree

❑ agree

❑ neutral

❑ disagree

❑ strongly disagree

General questions:

- How many minutes per day have you spent for this study?

- The time spent on this study was to much.

❑ strongly agree

❑ agree

❑ neutral

❑ disagree

❑ strongly disagree

- Operating the CAir-desk was difficult for me.

❑ strongly agree

❑ agree

❑ neutral

❑ disagree

❑ strongly disagree

- Do you consider [insert device] as user-friendly?

❑ Spirometer

❑ Everion-Armband

❑ FitBit-Armband

❑ Fragebogen

❑ Hustenaufzeichnung

❑ Foobot (Luftqualitätmessung)

❑ Sputum Charakterisierung

❑ Bedienung der Apps

- I would be willing to take part in this study for a longer time period.

❑ strongly agree

yes

❑ agree

❑ neutral

❑ disagree

no

❑ strongly disagree

Further comments:
