# Supplementary material for: Multisensory Home-Monitoring in Individuals With Stable Chronic Obstructive Pulmonary Disease and Asthma: Usability Study of the CAir-Desk
Source: JMIR Hum Factors. 2022 Feb 16;9(1):e31448. doi: 10.2196/31448 (PMC8892320; doi:10.2196/31448)
Supplement: Multimedia Appendix 2 [file humanfactors_v9i1e31448_app2.docx]

**Multimedia Appendix 2. Asthma symptom burden questionnaire.**

**Daily Asthma symptoms questionnaire**

- Did you experience Asthma related symptoms today?

❑ No

❑ light symptoms

❑ medium symptoms

❑ severe symptoms

- Did you experience Asthma related symptoms last night?

❑ No

❑ light symptoms

❑ medium symptoms

❑ severe symptoms

- Did you experience an Asthma attack today?

❑ No

❑ Yes

- Did you have to reschedule one of your activities due to Asthma related symptoms today?

❑ No

❑ Yes

- Did you have to stay away from work today due to Asthma related symptoms?

❑ No

❑ Yes

- How often did you have to use your Asthma rescue medication today?

❑ [insert number]

- Did you adhere to your medication prescription today?

❑ No

❑ Yes

❑ I do not have any medication prescribed
